# The Community Navigator Study: Results from a feasibility randomised controlled trial of a programme to reduce loneliness for people with complex anxiety or depression

**DOI:** 10.1371/journal.pone.0233535

**Published:** 2020-05-29

**Authors:** Brynmor Lloyd-Evans, Johanna Frerichs, Theodora Stefanidou, Jessica Bone, Vanessa Pinfold, Glyn Lewis, Jo Billings, Nick Barber, Anjie Chhapia, Beverley Chipp, Rob Henderson, Prisha Shah, Anna Shorten, Maria Giorgalli, James Terhune, Rebecca Jones, Sonia Johnson

**Affiliations:** 1 Division of Psychiatry, University College London, London, United Kingdom; 2 The McPin Foundation, London, United Kingdom; 3 Camden and Islington NHS Foundation Trust, St Pancras Hospital, London, United Kingdom; Washington State University, UNITED STATES

## Abstract

**Background:**

Loneliness is common among people with mental health problems and predicts poorer recovery from depression and anxiety. Needs for support with loneliness and social relationships are often under-addressed in mental health services.

The Community Navigator programme was designed to reduce loneliness for adults (aged 18 and above) with complex depression or anxiety who were using secondary mental health services. Acceptability and feasibility of the programme and a trial evaluation were tested in a feasibility randomised controlled trial with qualitative evaluation.

**Methods:**

Forty participants with depression or anxiety using secondary mental health services were recruited from mental health services in two London sites and randomised to receive: the Community Navigator programme over six months in addition to routine care (n = 30); or routine care (n = 10). Measures of loneliness, depression, other clinical and social outcomes and service use were collected at baseline and six-months follow-up. Levels of engagement in the programme and rates of trial recruitment and retention were assessed. Programme delivery was assessed through session logs completed by Community Navigators. The acceptability of the programme was explored through qualitative interviews (n = 32) with intervention group participants, their family and friends, programme providers and other involved staff.

**Results:**

Forty participants were recruited in four months from 65 eligible potential participants asked. No one withdrew from the trial. Follow-up interviews were completed with 35 participants (88%). Process records indicated the programme was delivered as intended: there was a median of seven meetings with their Community Navigator (of a maximum ten) per treatment group participant. Qualitative interviews indicated good acceptability of the programme to stakeholders, and potential utility in reducing loneliness and depression and anxiety.

**Conclusions:**

A definitive, multi-site randomised controlled trial is recommended to evaluate the effectiveness and cost-effectiveness of the Community Navigator programme for people with complex anxiety and depression in secondary mental health services.

## Background

Psychological and pharmacological interventions are ineffective, or only partially effective, for many people with severe anxiety or depression [1–4], of whom only those with the most complex needs or vulnerabilities are typically supported in secondary mental health services [5].

Loneliness, defined as the negative feeling deriving from the subjectively perceived gap between desired and actual social relationships [6] is common among people with mental health problems. Up to 40% of people with depression feel lonely most of the time [7], and people with mental health problems have a tenfold increase in the odds of being lonely, compared to the general population [8]. Being lonely predicts poorer recovery from anxiety and depression [9]. Programmes to reduce loneliness therefore hold promise as a means of improving social and clinical outcomes for people with depression or anxiety, including those with severe and long-term illness using secondary community mental health services, i.e. complex depression or anxiety. However, effective interventions for loneliness in people with mental health problems have yet to be established [10, 11], and social relationships and loneliness are often not addressed in mental health services [12].

The Community Navigator study sought to address these gaps in knowledge and service provision by developing and testing a socially-focused programme to reduce loneliness for people with complex depression or anxiety. The programme and its development are described more fully in the previously published trial protocol paper [13]. Here, we report the results of a feasibility randomised controlled trial of the Community Navigator programme as an addition to standard care from secondary mental health services, with an accompanying qualitative evaluation. We aimed to test the feasibility of programme delivery, trial procedures and recruiting and retaining participants in a randomised controlled trial. Through qualitative interviews, we explored the acceptability of the programme to participants, providers and other stakeholders.

## Method

### Trial methods

The protocol for this trial was prospectively registered (ISRCTN10771821). Its reporting in this paper adheres to CONSORT guidance [14]: a CONSORT checklist is provided in the data supplement (Additional S1 Checklist).

### Setting

The trial was conducted in two NHS Trusts. In one inner-London site, we recruited participants from a secondary mental health service for people with depression, anxiety, post-traumatic stress disorder and related non-psychotic disorders. In one outer-London site, we recruited participants with a primary diagnosis of depression or anxiety from four Community Mental Health Teams (which replaced a single dedicated service for people with complex anxiety or depression in a local service reorganisation at the time the trial started). Both areas were ethnically diverse, and included areas of affluence and of high economic deprivation.

### Participants

We recruited 40 trial participants, n = 20 at each site. Participants were required to meet a minimum threshold score for loneliness of two points on the six-item De Jong Gierveld loneliness scale [15] in initial screening. Participants were also required to be aged 18 or over and currently on the caseload of a secondary mental health service for people with depression or anxiety. People were excluded if they lacked decision-making capacity to consent to take part in the trial, were currently using mental health inpatient or crisis services, were assessed by the clinical team as posing a risk of harm to others such that meetings with researchers or a Navigator were not recommended, or could not communicate in English.

### Randomisation

Following baseline data collection, participants were individually randomised to the Community Navigators programme plus usual care, or usual care, in a 3:1 ratio using block randomisation stratified by site. The sample size and allocation ratio were chosen to be sufficient to assess the feasibility of trial recruitment and retention while maximising learning from delivering the programme to a substantial number of participants. Randomisation took place following baseline data collection. Group allocations were determined by an independent statistician at University College London, using a computer-generated allocation sequence. Following randomisation, participants, programme providers and researchers were not blind to participants’ allocation.

### The intervention

The Community Navigators programme was co-produced by the study working group, including members with lived experience, practitioners and academic researchers (see Additional S1 File). The programme comprised up to ten, hour-long meetings with a Community Navigator and access to up to three group sessions over a six month period. The timing of meetings was negotiated flexibly and meetings took place in participants’ homes, NHS premises, or in the community, as the participant preferred. A budget of up to £100 per participant was available, to be spent on activities designed to develop or enhance social connections and contact with others, as agreed with the Community Navigator.

The programme had three main components. First, the Community Navigator used a social network mapping tool, adapted from a previous study [12, 16] to map out people, places and activities which were important to the participant, and identify their current interests and relationships, and potential areas for new social activity, or means to strengthen existing connections with others. Second, the Community Navigator helped participants develop a “Connections Plan” which identified goals to increase connectedness and social relationships, and steps to achieve these. The Community Navigator then offered practical help or support in achieving these goals, including: locating social activities and groups which met the person’s interests and social identities; planning travel or accompanying a participant to a new social group; normalising and problem-solving setbacks or challenges. Third, the Community Navigators organised three group meet-up sessions available to all receiving the programme, which offered people a chance to meet each other, initiate friendships, and share experiences of the programme and recommendations about local groups and social opportunities.

Community Navigators were not required to have mental health professional training or qualifications, but were recruited on the basis of excellent interpersonal skills, awareness through personal or work experience of the challenges faced by people with serious mental illness, excellent knowledge of their local community and some previous work experience of supporting social inclusion and helping people develop social connections. Community Navigators were given five days’ initial training, delivered by members of the study co-production group, including study researchers, members with lived experience of mental health problems, and practitioners from the participating mental health teams. Training involved: familiarisation with and practice using the study network mapping and goal planning tools; guidance in using a solution-focused approach to supporting participants [17], focusing on their strengths and what participants can do to develop social connections; information about the nature of the patient group and mental health service context for the programme, including how to respond to and report concerns about participants’ safety or risks. A one-day induction in each clinical service was also provided, and three days’ “top-up” training, including a one-day workshop on coaching skills [18] and experiential peer-learning involving discussing challenges and ways of working. Fortnightly group supervision was provided to the Community Navigators by experienced social work and occupational therapy practitioners from the participating mental health services. The programme, its development and theoretical basis are described more fully in the published protocol paper [13]. The programme manual is available on the study website [19].

Participants in the treatment and control groups continued to receive standard care from secondary mental health services during the study period. This involved provision of a planned package of care comprising some or all of: meetings roughly monthly with a “care coordinator” i.e. a qualified mental health practitioner from the team such as a nurse or a social worker; appointments with a psychiatrist as required; and access to additional support from a psychologist on referral, if indicated.

### Measures

Socio-demographic information was collected from participants at baseline interview. The following outcomes relating to social contact or activity were measured for all participants at baseline and six-month, end-of-treatment follow up interviews:

- Loneliness, using the 11-item De Jong Gierveld scale [20], which yields a total score and social and emotional loneliness subscale scores
- Social network, using the 6-item Lubben Social Network Scale [21], which measures both the frequency and perceived quality of contact with family and friends
- Perceived social capital, using the 27-item Resource Generator UK measure [22]
- Activity, using the Time Budget Diary [23], which measures self-reported activity during the previous week. (This measure was adapted for the study to distinguish activities with others from those done alone.)

At baseline and six-month follow up, clinical outcomes were assessed using the following self-report scales:

- Depression, using the Patient Health Questionnaire (PHQ-9) measure [24]
- Anxiety, using the Generalised Anxiety Disorder (GAD-7) measure [25]
- Wellbeing, using the 14-item Warwick Edinburgh Mental Wellbeing Scale (WEMWBS) [26]

Health-related quality of life was assessed, also at baseline and six-month follow up, using the 10-item Recovering Quality of Life Questionnaire (REQOL) measure [27], which has been designed for mental health populations, and the five item EuroQol (EQ-5D-3L) measure [28]. Information was collected from NHS patient records about participants’ diagnoses at baseline, and about use of inpatient, crisis and community services for two time periods: the six months prior to baseline and during the six month study period. Information about participants’ use of social care services was similarly sought from Local Authority social care records.

Community Navigators were asked to complete a brief session log designed for the study after each meeting with a participant. These logs described the location of the meeting, whether any other people were involved, and the types of support offered, from a list of ten categories relevant to the manualised programme. As a reliability check for this data, a researcher sought feedback from each participant after two randomly selected meetings, using the same brief log.

### Procedures

Potential participants were screened for eligibility and initially contacted by clinical staff from participating services. A study researcher then contacted those who expressed an interest by phone to conduct loneliness screening and provide further information about the study. An information sheet was provided and discussed and written consent obtained, including willingness to be contacted regarding a qualitative interview. Written consent was confirmed before 6-month follow-up interviews.

Due to limited study resources, researchers collecting outcomes data could not be blinded. The study researcher contacted the independent statistician to randomise participants following baseline interviews; then contacted participants and their mental health teams to let them know which group they were allocated to. The study researcher contacted Community Navigators and participants to collect process data and to arrange 6-month follow-up interviews, and contacted NHS and Local Authority Informatics Teams to collect service use data retrospectively. Baseline and follow-up participant interviews were conducted face-to-face. Data collected on paper forms were securely stored at University College London, and entered into a secure database.

All participants were recruited were recruited within a four month period from April to July 2017. Six-month follow up interviews were completed between October 2017 and January 2018.

### Analysis

Feasibility and acceptability of the programme was assessed by intervention take up and retention rates among treatment group participants. Three meetings with a Community Navigator was set as a minimum threshold for treatment per protocol. Community Navigators’ session logs were used to assess the extent to which the programme was delivered as intended, i.e. how many participants had received key elements of the programme (visual mapping of their social world and development of a written plan to develop social connections), and the extent of its community focus (the proportion of meetings taking place outside people’s homes or health service premises, and the proportion of meetings involving social contact with other people). The number of adverse events for participants from recruitment to six-month follow-up was also reported, and whether any were assessed as study-related.

Feasibility of the trial was assessed by the proportion of eligible participants recruited, the length of time needed to recruit 40 participants, and attrition from the study: the proportion of participants retained in the study and who contributed follow up data. The quantity of missing data for trial outcome measures was also reported.

Participant characteristics at baseline and health outcomes at baseline and follow up were summarised separately by study arm. Loneliness and depression were identified as candidate primary outcomes for a future definitive trial: for these outcomes, we planned to estimate a baseline adjusted treatment effect and accompanying confidence interval using linear regression. The analysis was performed using Stata v14.

### Qualitative evaluation

#### Participants

We sought to recruit 20 participants from the trial; the three Community Navigators who delivered the programme, and ten other stakeholders who were sampled to include the Community Navigators’ supervisors and participants’ care coordinators from participating mental health teams, and involved family or friends who had met the Community Navigator at least once.

#### Measures

Separate topic guides were developed for semi-structured interviews with: participants, Community Navigators, and other stakeholders. These explored experiences of the programme, perception of any benefits and how they were achieved, barriers to engagement, and suggestions for improving the programme.

#### Procedures

Participants were contacted by a researcher and a separate information sheet was provided and written consent to participate was obtained. All participants from the treatment group were invited to take part in the qualitative interview. The researcher identified themselves to trial participants as a peer researcher with lived experience of mental health problems. Involved family and mental health staff were identified as potential interviewees through discussion with participants and Community Navigators. Interviews were audio-recorded, then transcribed. Transcripts were uploaded to Nvivo11 software for analysis.

#### Analysis

In an initial stage of analysis reported in this paper, a thematic analysis approach was used [29], with text coded into seven deductively-derived themes describing different domains of acceptability, based on an existing conceptual framework [30]. Participants’ suggestions for improvements to the programme were coded separately and are also reported here. Analysis was led by the two study researchers, with input from some members of the study team and coproduction working group, including lived experience and practitioner perspectives.

## Results

**Fig 1:**
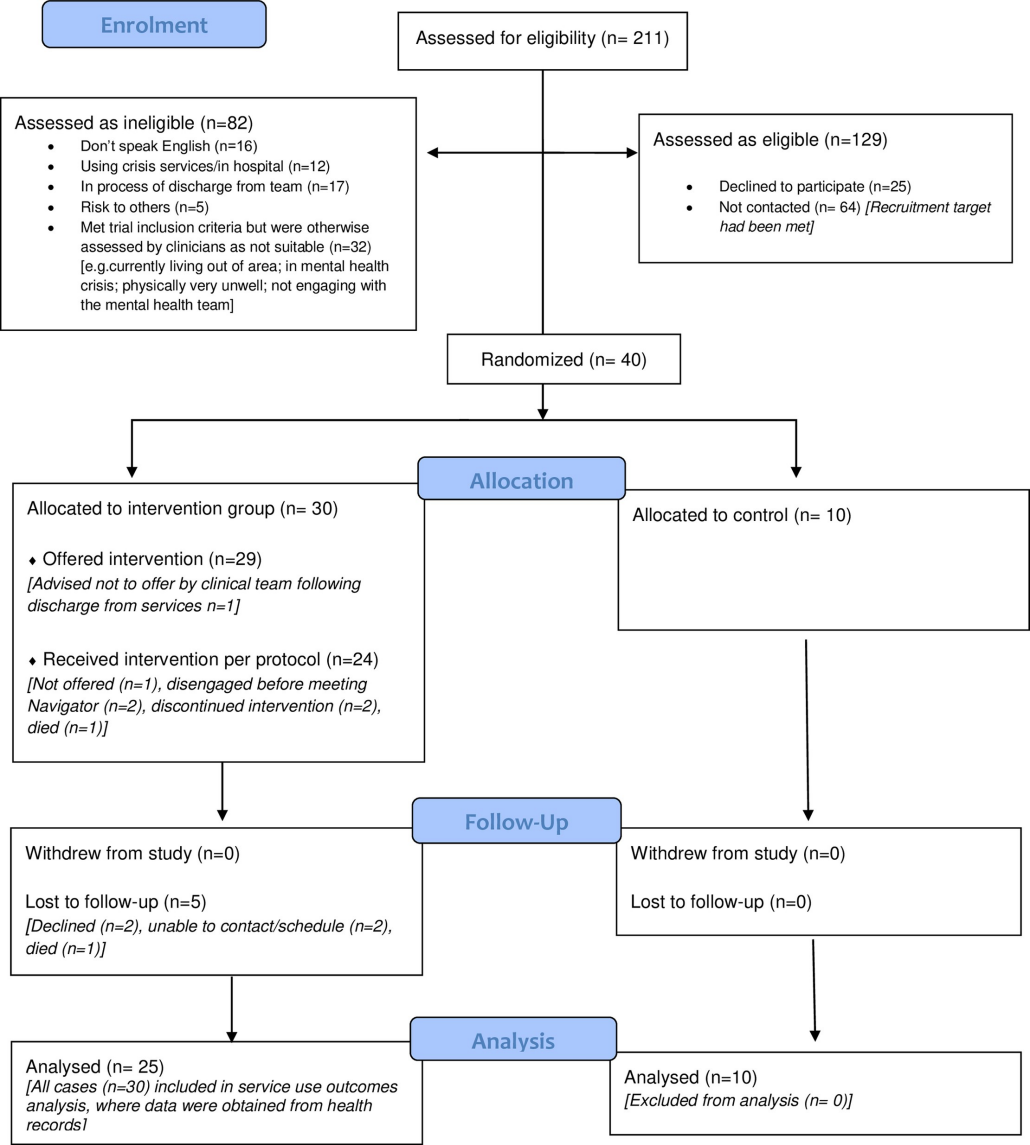
Community navigator feasibility trial consort diagram.

Fig 1 shows the recruitment and retention of trial participants. Of 211 people screened for the study, 129 (61%) were assessed as eligible potential participants. Sixty five of these potential participants were contacted about the study before our recruitment target was met: 40/65 (62%) agreed to take part and were recruited within four months. All potential participants who expressed an interest in taking part in the study met the study threshold score for loneliness: no one was excluded as not lonely. None of the 40 participants withdrew from the study, although one participant died during the study period. Follow-up interviews at six months after trial entry were completed with 35 of the 40 participants (88%).

The socio-demographic characteristics of trial participants are described in Table 1. Nearly two thirds of participants were from White ethnic groups (just under half were White British); nearly three quarters were female; and their mean age was 43 (range 19–69). Nearly half of participants lived alone; only one participant was married or cohabiting and only two were in paid employment. 80% (n = 32) had a primary diagnosis of anxiety or depression.

**Table 1:** Participants’ socio-demographic characteristics at baseline.

Characteristic	Intervention (N = 30)	Control (N = 10)	All participants (N = 40)
**Sex–**N (%)			
Male	6 (20)	5 (50)	11 (28)
Female	24 (80)	5 (50)	29 (73)
**Age–**Mean (SD)	44.6 (13.4)	38.5 (11.8)	43.1 (13.1)
**Ethnicity**–N (%)			
White	17 (59)	8 (80)	25 (64)
Mixed/ Multiple ethnic groups	3 (10)	0 (0)	3 (8)
Asian/ Asian British	3 (10)	1 (10)	4 (10)
Black/ African/ Caribbean/ Black British	4 (14)	1 (10)	5 (13)
Other ethnic group	2 (7)	0 (0)	2 (5)
**Housing Situation–**N (%)			
Independent permanent accommodation	22 (73)	9 (90)	31 (78)
Independent temporary accommodation	5 (17)	1 (10)	6 (15)
Accommodation with staff support	3 (10)	0 (0)	3 (8)
**Living Situation–**N (%)			
Lives alone	14 (47)	5 (50)	19 (48)
Lives with other adults, no dependent children	9 (30)	3 (30)	12 (30)
Lives with dependent children	7 (23.3)	2 (20)	9 (23)
**Marital Status–**N (%)			
Single	18 (60)	7 (70)	25 (63)
Married or cohabiting	0 (0)	1 (10)	1 (3)
Separated or divorced	11 (37)	2 (20)	13 (33)
Widowed	1 (3)	0 (0)	1 (3)
**Employment/ Education Status–**N (%)			
Open Market Employment	0 (0)	2 (20)	2 (5)
Education, study or training	2 (6)	2 (20)	4 (10)
Voluntary or unpaid work	4 (13)	0 (0)	4 (10)
Full time Caring role	2 (6)	1 (10)	3 (8)
Other	22 (73)	5 (50)	27 (68)
**Primary Diagnosis–**N (%)			
F32-39 Mood (affective) disorders	12 (40)	3 (30)	15 (38)
F40-48 Anxiety disorders	13 (43)	4 (40)	17 (43)
Other disorders	5 (17)	3 (30)	8 (20)
**GP appointments in the 3 months prior to baseline—**Median (IQR)	4.5 (2.0 to 8.0)	2.0 (1.0 to 3.0)	(1.5 to 6.5)

SD = standard deviation IQR = Interquartile range

The denominator for percentages is the number of non-missing values.

Participants’ scores on outcome measures at baseline and 6-month follow-up are summarised in Table 2. Mean scores and standard deviation (SD) are presented for measures where data were normally distributed, and median scores and interquartile range (IQR) where data were skewed. Full results including subscale scores are provided in the Data Supplement (Additional S1 File). At baseline, participants’ mean depression score met the established threshold for severe depression [31]. Participants were also extremely lonely: median score at baseline on the De Jong Gierveld loneliness scale was 11, i.e. the maximum score on the measure.

**Table 2:** Participant outcomes: baseline and 6 month follow-up.

Outcomes	Baseline		6 month follow-up	
	Intervention (N = 30)	Control (N = 10)	Intervention (N = 25*)	Control (N = 10)
**Loneliness: De Jong-Gierveld (DJG) Loneliness Scale**				
Total Score—Median (IQR)	11.0 (10.0 to 11.0)	10.5 (9.0 to 11.0)	9.0 (8.0 to 11.0)	10.0 (7.0 to 11.0)
Social Loneliness subscale score	5.0 (5.0 to 5.0)	5.0 (4.0 to 5.0)	5.0 (4.0 to 5.0)	4.0 (4.0 to 5.0)
Emotional Loneliness subscale score	6.0 (5.0 to 6.0)	6.0 (5.0 to 6.0)	5.0 (4.0 to 6.0)	6.0 (4.0 to 6.0)
**Depression: Patient Health Questionnaire (PHQ-9)**				
Total score—Mean (SD)	21.6 (5.3)	21.1 (4.5)	16.4 (6.8)	18.8 (4.8)
**Anxiety: Generalized Anxiety Disorder Questionnaire (GAD-7)**				
Total score—Median (IQR)	19.0 (15.0 to 21.0)	16.0 (11.0 to 18.0)	14.0 (10.5 to 17.5)	13.5 (11.0 to 16.0)
**Wellbeing: Warwick-Edinburgh Mental Well-being Scale (WEMWBS)**				
Total score—Median (IQR)	26.5 (20.0 to 32.0)	30.0 (24.0 to 34.0)	29.5 (23.0 to 34.5)	31.0 (23.0 to 37.0)
**Social network: Lubben Social Network scale (LSNS6)**				
Total score—Median (IQR)	7.0 (4.0 to 9.0)	11.5 (9.0 to 15.0)	7.5 (6.0 to 11.0)	11.0 (6.0 to 15.0)
**Perceived social capital: Resource Generator UK (RGUK)**				
Total score—Median (IQR)	9.5 (5.0 to 12.0)	13.0 (8.8 to 18.3)	9.0 (6.0 to 12.3)	13.0 (6.5 to 22.3)
**Activity: Time Budget Diary (TBD)**				
Total score–Mean (SD)	32.7 (9.6)	38.4 (11.2)	36.9 (12.9)	35.0 (14.4)
**Recovering Quality of Life Questionnaire (ReQoL-10)**				
Total score—Median (IQR)	9.0 (4.0 to 14.0)	9.5 (5.0 to 15.0)	14.5 (8.0 to 19.0)	13.5 (10.0 to 19.0)
**EuroQol Health Questionnaire (EQ-5D-5L) Index value–**Mean (SD)	0.283 (0.40)	0.400 (0.24)	0.472 (0.33)	0.453 (0.236)
**Self-rated health using EQ-Visual Analogue Scale (EQ VAS)—**Median (IQR)	35.0 (29.0 to 50.0)	47.5 (30.0 to 50.0)	40.0 (30.0 to 60.0)	52.5 (35.0 to 60.0)
**Accepted for acute treatment in past 6 months (hospital or community crisis care**)–N (%)	6 (20)	0 (0)	5 (20)	1 (10)
**Days in acute care—**Median (IQR)	0.0 (0.0 to 0.0)	0.0 (0.0 to 0.0)	0.0 (0.0 to 0.0)	0.0 (0.0 to 0.0)
**Admitted to hospital–**N (%)	1 (3)	0 (0)	0 (0)	1 (10)
**Inpatient bed days–**Median (IQR)	0.0 (0.0 to 0.0)	0.0 (0.0 to 0.0)	0.0 (0.0 to 0.0)	0.0 (0.0 to 0.0)
**Community service kept appointments–**Median (IQR)	6.5 (3.0 to 11.0)	6.5 (1.0 to 17.0)	3.5 (2.0 to 10.0)	8.0 (1.0 to 10.0)
**Community service missed appointments–**Median (IQR)	0.0 (0.0 to 1.0)	0.0 (0.0 to 1.0)	0.0 (0.0 to 1.0)	1.5 (0.0 to 3.0)
**Missed one or more community service appointments–**N (%)	11 (44)	4 (44)	10 (33)	6 (60)

SD = standard deviation IQR = Interquartile range.

* Follow-up data from self-report questionnaires were collected for n = 25 treatment group participants and n = 10 control group participants. Service use data from health records were collected for all participants (n = 40). There were more missing data at baseline in the intervention than the control group for missed appointments (n = 5 vs n = 1; 17% vs 10%) and TBD (n = 3 vs n = 0; 10% vs 0%). For all other outcomes at baseline and among participants who completed the follow up interview there was a maximum of 1 or 2 missing values per study arm, mostly in the intervention group, and missing data did not exceed 10% in either arm. The denominator for percentages is the number of non-missing values.

Among completed interviews, levels of missing data were very low (see Additional S1 File). Loneliness in the intervention group fell from a median De Jong Gierveld Scale score of 11 at baseline to 9 at follow-up, and from 10.5 to 10 for control group participants. However, due to the highly skewed distribution of data for this outcome, an effect size for the intervention was not calculated. Mean depression scores on the PHQ-9 measure fell from 21.6 to 16.4 during the intervention period for participants in the treatment group, compared to a fall from 21.1 to 18.8 in the control group. The change in depression score from baseline to follow up in the treatment group exceeds the established five-point threshold for clinically meaningful change on PHQ-9 [31]. The adjusted mean difference between groups on PHQ-9 scores at follow up was -2.54, (95% confidence intervals: -6.53 to 1.44, p = 0.20). This indicates that the true effect of the intervention for depression lies somewhere between a large positive effect and a small negative effect.

For the 30 treatment group participants, the median number of meetings per participant with a Community Navigator was seven, and 12 participants (40%) attended at least one group meet-up. Twenty four of 30 intervention group participants (80%) met our pre-specified minimum threshold for treatment per protocol of at least three meetings with a Community Navigator. Session logs were completed for all 186 meetings between Community Navigators and participants. These logs indicate that all 24 participants who were treated per protocol completed network mapping, and 21 of these 24 set goals for their community connections plan. Sixty four percent of meetings with a Community Navigator took place at a community venue (not the participant’s home or NHS premises), and 37% of meetings involved social contact with other people as well as the Community Navigator. Only nine of 30 treatment group participants used funding from the Community Navigation budget (up to £100 per participant) to support social activity. Comparison of process data collected from Community Navigators’ session logs and the researcher’s phone interviews with participants indicated fair levels of agreement (72%) regarding the types of support provided (see Data Supplement–Additional S1 File).

Regarding routine care: participants in the treatment group met a member of the mental health team (not including the Community Navigator) a median of 3.5 times during the six month study period, with a median of 8 meetings during this period for control group participants. Seven participants (17.5%) were discharged from mental health services during the study period: four in the treatment group; three in the control group. Local government departments declined to facilitate access to participants’ social care records (i.e. records of non-health, statutorily-provided care), despite participants’ written consent for this, so we were unable to collect information about use of social care services.

There were two serious adverse events during the study. One participant in the intervention group died through suicide; one participant in the control group was admitted voluntarily to psychiatric hospital. Both events were independently assessed as not related to their participation in the study.

### Qualitative evaluation

Interviews were conducted with 19 participants from the intervention group, i.e. all those who could be contacted and then agreed to be interviewed. A further 13 interviews were conducted with the Community Navigators (n = 3); their supervisors (n = 3); participants’ care coordinators (n = 4), and involved family or friends of participants (n = 3). Findings regarding the acceptability of the programme are summarised for each domain of acceptability used in analysis, followed by suggested improvements for the programme. To maintain anonymity, all professional stakeholders are described as “staff member” in the included quotations. A fuller summary of acceptability themes, including their definitions, and additional illustrative quotations are available from the authors on reasonable request.

### Affective attitude: How an individual feels about an intervention

Nearly all those who received the programme had a positive experience of taking part, while other stakeholders felt it was a useful addition to mental health support.

“*It was wonderful*. *I really enjoyed the experience*, *it was amazing*. *I’d do it again if I could*. *I’d have another ten sessions and carry on with it*.*”* Participant SU14

“*I think it’s really excellent because I think particularly for our client group a lot of people we see here are really socially isolated*.*”* Staff member 6

Central to participants’ experience of the programme, was their relationship to the Community Navigator. Participants described feeling valued, cared about and understood, in contrast to some of their previous experiences of support. Participants valued being encouraged to make changes but not pressurised.

“*The only thing that comes across clearly is he actually does care*.*” Participant SU03*

“*She came across as a bubbly person and optimistic–like*, *‘You can do this*, *[participant]*.*’ I said*, *‘Can I*?*’ She went*, *‘Yes*, *you can*.*’ And I did*.*” Participant SU4*

In contrast, a single participant reported that the Community Navigator was too focused on trying to identify an activity of interest, before building a relationship.

### Burden: The perceived amount of effort required to participate

Several aspects of the programme were perceived to be effortful. Participants described being active or making social contact with others as difficult when they felt low or tired, or highly anxious. They described a conflict between wanting more social contact and concern about the additional pressure that maintaining relationships might bring.

“*I have claustrophobia and I have agoraphobia… I knew I was going to go through quite a bit of suffering to manage to stay for three hours in the class*.*”* Participant SU17

“*The old me was quite good at that*, *making new connections but following through is always so hard for me that it just feels pointless because it feels like another thing where you've let yourself down or someone else*.*”* Participant SU13

However, people explained how pushing themselves to face things they found challenging sometimes resulted in a sense of achievement and increased confidence.

“*The first group session I did find*, *"Am I going to be alright*? *There's lots of new people*, *new faces … I actually did consider not going*. *Then I thought*, *"No*, *make yourself go*. *If you don't then you're not even trying*.*" So I did*, *I made myself go and it's the best thing I did*.*”* Participant SU8

### Ethicality: The intervention’s fit with an individual’s value system

There were few direct references to ethical concerns. Staff and friends/family in particular suggested that the programme fitted with interviewees’ personal and professional beliefs that mental health services should address participants’ broader life needs, as well as their symptoms.

“*We’re thinking a lot about people’s medication and their symptoms… but that’s not all that you need to have a good life*, *is it*? *You need to have that quality of life as well*, *and it’s the first time that it’s been sort of particularly seriously addressed by any team that I’ve worked in”* Staff Member 5

“*This is a study that can actually give you the purpose*. *It's not plying you with medication or putting you on programmes that might work*, *it's giving you purpose that you design so that's really important*.*”* Friend/family member 3

### Intervention coherence: Participants’ understanding of the intervention and how it works

Apart from one participant and one staff member, all other interviewees were able to describe what the programme was about. Some described the programme in terms of direct support from a Community Navigator with going to social activities; others focused on the longer-term goals of the programme in terms of improving people’s social connectedness and mental health through the support in the programme.

“*I've been through a lot of low times with depression and anxiety*. *It's left me disengaging myself with everyone*, *like my friends and my family and just wanting to stay in a lot and be on my own*. *I just thought that this was the perfect study to get me to interact with people but also to do something that I enjoy*.*”* Participant SU10

Two key elements to the programme were identified: providing dedicated time and space to focus on social connections; and the Community Navigators’ focus on moving forward and doings things, in contrast to other mental health roles, which can focus more on problems and the past.

“*It was really different from counselling where you turn up and talk about how things have been and dwell on how things made you feel*.*”* Participant SU11

“*I think the navigators bring the ability to be able to work more regularly and more specifically on reducing social isolation*. *Whereas care coordinators may try to do that but often their role may bring them away from that*.*”* Staff member 3

### Opportunity cost: The extent to which benefits, profits, or values must be given up to engage in the intervention

Few data were coded as opportunity cost. However, one example was a Community Navigators’ supervisor, who described the significant time commitment and benefits of being involved in the programme.

“*Obviously I had to make more time…but it wasn’t a negative impact*, *it was a positive impact*. *It brought something alive in the team*.*”* Staff member 1

### Perceived effectiveness: Whether the intervention is perceived as likely to achieve its purpose

Some participants described how the programme had helped them considerably, although others felt that the programme was not sufficiently long to wholly address the longstanding nature of people’s mental health problems and loneliness.

“*I think it leaves you needing more help*. *It leaves you*, *okay*, *I’ve opened up these avenues now… but there’s no follow-up*. *It ends and then you’re…seeing somebody six*, *seven times is not enough*.*”* Participant SU17

“*I’ve reconnected with friends from secondary school*. *Yes*, *I’ve reconnected with a lot of people and I haven’t been feeling quite so lonely at all*.*”* Participant SU10

“*I would still be moping around*, *depressed*, *with nothing to look forward to*. *Yes*, *so it helped me a great deal this*, *yes*.*”* Participant SU15

Some respondents described how the programme might not resolve all difficulties, but could initiate positive longer-term change. These building blocks identified from the programme included: being more aware of social opportunities locally, feeling more comfortable interacting with others, and starting to attend regular groups or courses.

“*[community navigator] has helped me in the fact that she's made me try to see some people differently to what I may initially*… *not to just initially cut everybody off from the start without giving it a chance and seeing whether we would get on*.*”* Participant SU01

The timing of the programme was identified as a key consideration. Its impact could be limited if participants faced complex life factors during the intervention period.

“*I think that there were developments along the way in her personal life that made it difficult to fully engage…she could have got a lot more out of it and realises that*, *if it had come at a different time for her*.*”* Friend/family member 3

### Self-efficacy: Participants’ confidence that they can perform the behaviour required to participate in the intervention

There was overlap in the data coded under self-efficacy and burden. Social interaction was perceived as hard and an area where participants lacked confidence.

“*I need to be pushed…I want to do stuff*, *and then it’s like*, *in the morning I wanted to do this and this*, *after half an hour or an hour or two hours*, *I feel completely opposite*, *I don’t want to do anything*.*”* Participant SU09

“*She doesn't think people will like her*. *She doesn't have any confidence in her ability*. *She doesn't trust people either of course*. *So she won't meet them to find out whether they like her or not*.*”* Friend/Family member 3

However, interviewees also described how Community Navigators worked with them to find ways to make social engagement feel manageable, and how the presence of their Community Navigator had enabled them to face situations they would otherwise avoid.

“*She would try and get me to use the phone*, *but I used to panic…I did eventually do it myself*. *She wrote things down for me to say on the phone*, *for me to explain*.*”* Participant SU14

“*I went with [community navigator]…which was good because the feelings I had*, *I just wanted to bolt*. *I panicked because there was so many people around*. *If I'd gone by myself*, *I would never have got as far*.*”* Participant SU01

### Suggested improvements

The main improvements suggested by some participants, Community Navigators and other stakeholders were a longer period of support and more sessions. Several interviewees felt that the navigators programme was not long enough and described how life circumstances and scheduling conflicts limited service users’ engagement.

“*The other problem was we had to use all our sessions up by December*. *That made it really difficult because it takes time to get things rolling*, *it takes time for courses to start and enrol on them and try them out…It was just a few sessions and then it was finished*. *It's not long enough*.*”* Participant SU17

“*It would have been good to do this for a bit longer so that they could have spread out the sessions a little bit more*, *so that if they’d been not well or one person went on holiday for six weeks over the summer … so it’s been difficult to get any momentum going*.*”* Staff member 2

Some interviewees suggested that Community Navigators could potentially be more effective if they were to work more closely with the client’s mental health team.

“*I think that coming together with the Community Navigator a bit more and giving them more insight into our clients and how to approach our clients in some respects”* Staff member 4.

## Discussion

### Main findings

Our feasibility trial demonstrates that a socially-focused Community Navigators Programme designed to reduce loneliness can be delivered as intended for people with complex depression or anxiety, in secondary mental health services as an addition to standard care. Qualitative feedback and session logs indicate that the programme focused as planned on social relationships. Support from a Community Navigator to review their social world, set goals to develop social connections and then to act on them appeared to help participants to increase knowledge about opportunities for social interaction, access new social groups and activities, and develop or regain positive social identities and connections with others.

The high take up rate for the study (40 of 65 people asked) suggest high levels of interest among patients for support of this type. The good rate of retention in the programme and its positive appraisals in qualitative interviews suggest it is acceptable to participants, and to mental health staff and participants’ families. The high rates of trial recruitment, retention and data completeness suggest that evaluation of the Community Navigators programme in a randomised controlled trial is feasible. The feasibility trial was not intended to and did not establish the effectiveness of the programme, but quantitative and qualitative results are promising and support further evaluation of its effectiveness.

### Strengths and limitations

The study took place in one inner London and one outer-London NHS Trust. Findings are not necessarily generalizable to other contexts, e.g. rural areas. Although we sought to provide the programme as an addition to multidisciplinary care from secondary mental health services, seven of the forty trial participants were discharged from mental health services during the study period.

Blinding of researchers was not possible due to limited study resources, although this could be planned for a full trial. Blinding of participants and programme providers was not possible due to the nature of the study intervention. Outcomes data were only collected from participants at a six month time point: rates of participant retention in a trial beyond this end-of-treatment time point are therefore uncertain. In other respects, this study was designed and successfully conducted like a definitive trial in miniature.

Process recording through Community Navigators’ session logs is vulnerable to possible reporting bias. Collecting process data by video or audio-recording sessions is not practical for this intervention, where most meetings took place in public community settings and was not recommended as acceptable to participants by our lived experience working group members. Brief phone interviews with participants corroborated session logs to an extent, with fair levels of agreement, but some participants found categorising different types of support confusing. A simpler schedule for obtaining feedback from participants about the content of care may be required in future studies.

Qualitative data analysis for this paper focused on the acceptability of the programme. An additional, inductive analysis of data will further explore participants’ experience of loneliness and perceived mechanisms of effect of the programme. This will be reported separately.

### Implications for research

The main implication of this successful feasibility trial is that a definitive evaluation of the effectiveness and cost-effectiveness of the Community Navigators programme is recommended, through a multi-site, randomised controlled trial.

A manual for the programme, and a training programme and support and supervision structures have been developed. The programme is therefore tested and ready for evaluation in a full trial. Two aspects of the programme as currently structured warrant consideration regarding possible adaptation. First, the group meet-ups organised in addition to individual support from a Community Navigator were attended by only a minority of participants. They were valued by those participants who did attend however, and we consider it is unlikely that any single group format could engage all study participants. Second, several participants in qualitative interviews expressed a wish for more sessions or a longer duration of support from the Community Navigator. We are mindful that increasing the number of meetings would increase the cost of the programme and reduce how many people it could be offered to in routine settings. Increasing the number of sessions or the duration of the programme risks moving its focus more to an ongoing relationship with the Community Navigator, rather than providing time-limited support for participants to connect with others and forge social relationships in their community.

Regarding trial procedures, our study suggests the De Jong Gierveld Loneliness Scale [24] may not be the most suitable measure of loneliness in this setting. It had a ceiling effect for our trial’s severely depressed, extremely lonely participant group, more than half of whom obtained the maximum score on the measure at baseline. Its scoring system may also not be very sensitive to change for people who are extremely lonely. Respondents are offered a choice of three Likert scale responses when completing the questionnaire, but these are then dichotomised to score each item. So, for example, if a participant’s response to the statement “There are enough people I feel close to” changed from “no” at baseline to “more or less” at follow-up, this would be scored the same at both time points, even though it might reflect a personally very meaningful change in someone’s experienced loneliness. Other established measures of loneliness, such as the UCLA Loneliness Scale [32] may be preferable as a trial outcome measure. In a future trial, we would also consider using a self-report measure such as the Client Service Receipt Inventory [33] to collect information about use of community services, given our inability to access participants’ records held by Local Authorities.

### Implications for practice

Our study suggests that many people with complex depression and anxiety receiving care from secondary mental health services are extremely lonely. It suggests that additional support to improve social connections and reduce loneliness would be welcomed by many and meet a gap in current service provision. In the current absence of interventions with established evidence of effectiveness for reducing loneliness in this clinical group [10, 11], the Community Navigator programme offers a preliminarily-tested programme for reducing loneliness for people with complex depression or anxiety, with a programme manual and training manual which are available for reference or use in the NHS and other health settings.

## Supporting information

S1 Checklist CONSORT checklist_Community Navigator Study. (DOC)

S1 File Full study results Community Navigator Study. (DOCX)
