# Supplementary material for: The Community Navigator Study: Results from a feasibility randomised controlled trial of a programme to reduce loneliness for people with complex anxiety or depression
Source: PLoS One. 2020 May 29;15(5):e0233535. doi: 10.1371/journal.pone.0233535 (PMC7259554; doi:10.1371/journal.pone.0233535)
Supplement: S1 Checklist — (DOC) [file pone.0233535.s001.doc]

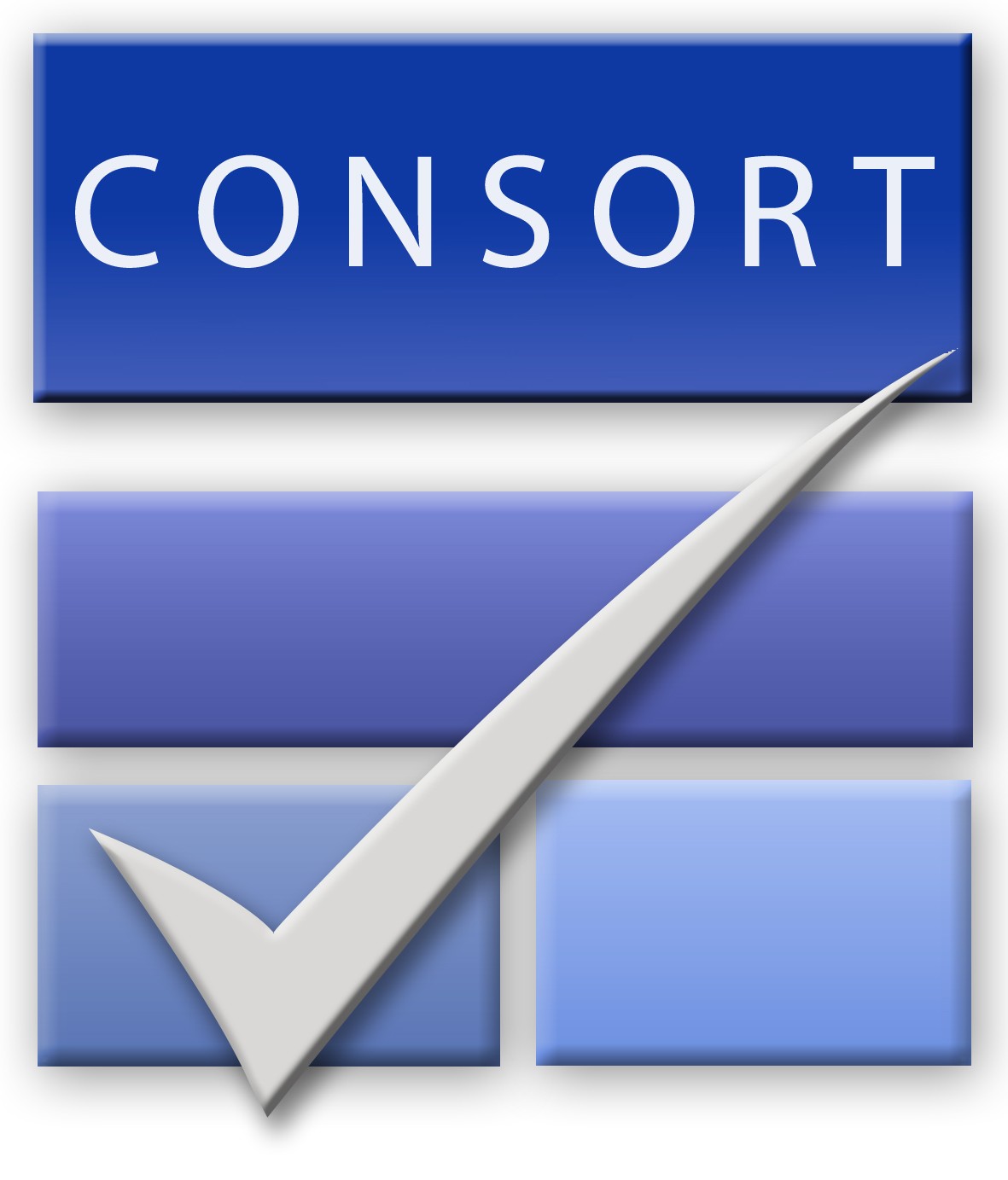
CONSORT 2010 checklist: Community Navigator Feasibility Trial

| Section/Topic | Item No | Checklist item | Reported |
| --- | --- | --- | --- |
| Title and abstract | | | |
|  | 1a | Identification as a randomised trial in the title | Study title |
|  | 1b | Structured summary of trial design, methods, results, and conclusions (for specific guidance see CONSORT for abstracts) | Abstract |
| Introduction | | | |
| Background and objectives | 2a | Scientific background and explanation of rationale | Background section |
|  | 2b | Specific objectives or hypotheses | Aims section |
| Methods | | | |
| Trial design | 3a | Description of trial design (such as parallel, factorial) including allocation ratio | Methods – randomisation section |
|  | 3b | Important changes to methods after trial commencement (such as eligibility criteria), with reasons | N/A |
| Participants | 4a | Eligibility criteria for participants | Methods – Participants section |
|  | 4b | Settings and locations where the data were collected | Methods – setting section |
| Interventions | 5 | The interventions for each group with sufficient details to allow replication, including how and when they were actually administered | Methods – intervention section with references to protocol paper and intervention manual |
| Outcomes | 6a | Completely defined pre-specified primary and secondary outcome measures, including how and when they were assessed | Feasibility outcomes specified – methods, analysis section  Outcome measures of effectiveness used in feasibility trial specified – methods, measures section |
|  | 6b | Any changes to trial outcomes after the trial commenced, with reasons | N/A |
| Sample size | 7a | How sample size was determined | Methods – randomisation section |
|  | 7b | When applicable, explanation of any interim analyses and stopping guidelines | N/A |
| Randomisation: |  |  |  |
| Sequence generation | 8a | Method used to generate the random allocation sequence | Methods – randomisation section |
|  | 8b | Type of randomisation; details of any restriction (such as blocking and block size) | Methods – randomisation section |
| Allocation concealment mechanism | 9 | Mechanism used to implement the random allocation sequence (such as sequentially numbered containers), describing any steps taken to conceal the sequence until interventions were assigned | Methods – randomisation section |
| Implementation | 10 | Who generated the random allocation sequence, who enrolled participants, and who assigned participants to interventions | Sequence generation – methods, randomisation section  Participant enrolment and assignment – methods, procedures section |
| Blinding | 11a | If done, who was blinded after assignment to interventions (for example, participants, care providers, those assessing outcomes) and how | Methods – randomisation section |
|  | 11b | If relevant, description of the similarity of interventions | N/A |
| Statistical methods | 12a | Statistical methods used to compare groups for primary and secondary outcomes | Methods, analysis section |
|  | 12b | Methods for additional analyses, such as subgroup analyses and adjusted analyses | N/A |
| Results | | | |
| Participant flow (a diagram is strongly recommended) | 13a | For each group, the numbers of participants who were randomly assigned, received intended treatment, and were analysed for the primary outcome | Figure 1, CONSORT diagram |
|  | 13b | For each group, losses and exclusions after randomisation, together with reasons | Figure 1, CONSORT diagram |
| Recruitment | 14a | Dates defining the periods of recruitment and follow-up | Results, first paragraph |
|  | 14b | Why the trial ended or was stopped | N/A |
| Baseline data | 15 | A table showing baseline demographic and clinical characteristics for each group | Table 1 |
| Numbers analysed | 16 | For each group, number of participants (denominator) included in each analysis and whether the analysis was by original assigned groups | Table 2 |
| Outcomes and estimation | 17a | For each primary and secondary outcome, results for each group, and the estimated effect size and its precision (such as 95% confidence interval) | Table 2 for descriptive results  Results, fourth paragraph for estimated effect size for depression scores |
|  | 17b | For binary outcomes, presentation of both absolute and relative effect sizes is recommended | N/A |
| Ancillary analyses | 18 | Results of any other analyses performed, including subgroup analyses and adjusted analyses, distinguishing pre-specified from exploratory | N/A |
| Harms | 19 | All important harms or unintended effects in each group (for specific guidance see CONSORT for harms) | Results, paragraph 7 |
| Discussion | | | |
| Limitations | 20 | Trial limitations, addressing sources of potential bias, imprecision, and, if relevant, multiplicity of analyses | Discussion, limitations section |
| Generalisability | 21 | Generalisability (external validity, applicability) of the trial findings | Discussion, limitations section |
| Interpretation | 22 | Interpretation consistent with results, balancing benefits and harms, and considering other relevant evidence | Discussion, main findings and implications for research section |
| Other information | | |  |
| Registration | 23 | Registration number and name of trial registry | Abstract and method, first paragraph |
| Protocol | 24 | Where the full trial protocol can be accessed, if available | Reference to published protocol paper - Introduction |
| Funding | 25 | Sources of funding and other support (such as supply of drugs), role of funders | Funding acknowledgement at the end of the paper |

*We strongly recommend reading this statement in conjunction with the CONSORT 2010 Explanation and Elaboration for important clarifications on all the items. If relevant, we also recommend reading CONSORT extensions for cluster randomised trials, non-inferiority and equivalence trials, non-pharmacological treatments, herbal interventions, and pragmatic trials. Additional extensions are forthcoming: for those and for up to date references relevant to this checklist, see [www.consort-statement.org](http://www.consort-statement.org/).
