# Supplementary material for: The Community Navigator Study: Results from a feasibility randomised controlled trial of a programme to reduce loneliness for people with complex anxiety or depression
Source: PLoS One. 2020 May 29;15(5):e0233535. doi: 10.1371/journal.pone.0233535 (PMC7259554; doi:10.1371/journal.pone.0233535)
Supplement: S1 File — (DOCX) [file pone.0233535.s002.docx]

**Community Navigator Study**

**Appendix 2: full study quantitative results and co-production summary**

**Contents**

|  | **Page** |
| --- | --- |
| **Trial quantitative results** | **2** |
| **Trial process evaluation** | **7** |
| **Adverse events** | **11** |
| **Coproduction in the study** | **12** |

**Trial Quantitative Results**

**Table 1. Community Navigator Trial: Participant characteristics**

| **Characteristic** | **Intervention  (N = 30)** | **Control (N = 10)** | **All participants**  **(N = 40)** |
| --- | --- | --- | --- |
| **Sex** – N (%)  Male  Female | 6 (20)  24 (80) | 5 (50)  5 (50) | 11 (28)  29 (73) |
| **Age** – Mean (SD) | 44.6 (13.4) | 38.5 (11.8) | 43.1 (13.1) |
| **Ethnicity** – N (%)  White  Mixed/Multiple ethnic groups  Asian/Asian British  Black/African/ Caribbean/ Black British  Other ethnic group | 17 (59)  3 (10)  3 (10)  4 (14)  2 (7) | 8 (80)  0 (0)  1 (10)  1 (10)  0 (0) | 25 (64)  3 (8)  4 (10)  5 (13)  2 (5) |
| **Housing Situation** – N (%)  Independent permanent accommodation  Independent temporary accommodation  Accommodation with staff support | 22 (73)  5 (17)  3 (10) | 9 (90)  1 (10)  0 (0) | 31 (78)  6 (15)  3 (8) |
| **Living Situation** – N (%)  Lives alone  Lives with other adults, no dependent children  Lives with dependent children | 14 (47)  9 (30)  7 (23.3) | 5 (50)  3 (30)  2 (20) | 19 (48)  12 (30)  9 (23) |
| **Marital Status** – N (%)  Single  Married or cohabiting  Separated or divorced  Widowed | 18 (60) 0 (0)  11 (37)  1 (3) | 7 (70)  1 (10)  2 (20)  0 (0) | 25 (63)  1 (3)  13 (33)  1 (3) |
| **Employment/Education Status** – N (%)  Open Market Employment  Education, study or training  Voluntary or unpaid work  Full time Caring role  Other | 0 (0)  2 (6)  4 (13)  2 (6)  22 (73) | 2 (20)  2 (20)  0 (0)  1 (10)  5 (50) | 2 (5)  4 (10)  4 (10)  3 (8)  27 (68) |
| **Diagnosis** – N (%)  F30-31 Bipolar Disorder  F32-39 Mood (affective) disorders  F40-48 Anxiety Disorders  F50-59 Behavioural syndromes  F60-69 Personality Disorders  F80-89 Developmental Disorders | 0 (0)  12 (40)  13 (43)  1 (3)  3 (10)  1 (3) | 1(10)  3 (30)  4 (40)  0 (0)  1(10)  1 (10) | 1 (3)  15 (38)  17 (43)  1 (3)  4 (10)  2 (5) |
| **GP Appointments the 3 months prior to baseline** - Median (IQR*) | 4.5 (2.0 to 8.0) | 2.0 (1.0 to 3.0) | 1. (1.5 to 6.5) |

The denominator for percentages is the number of non-missing values.(There was one missing response for ethnicity.)

*IQR=Interquartile range

**Table 2. Community Navigator trial participant outcomes: Baseline and 6 month follow-up**

| **Outcomes** | **Baseline** | | | **6 month follow-up** | |
| --- | --- | --- | --- | --- | --- |
|  | **Intervention**  **(N=30)** | **Control**  **(N=10)** | **All participants**  **(N = 40)** | **Intervention**  **(N = 25)** | **Control (N = 10)** |
| **Loneliness: De Jong Gierveld (DJG) Loneliness Scale**  Total score - Median (IQR)*  Social Loneliness subscale  Emotional Loneliness subscale | 11.0 (10.0 to 11.0)  5.0 (5.0 to 5.0)  6.0 (5.0 to 6.0) | 10.5 (9.0 to 11.0)  5.0 (4.0 to 5.0)  6.0 (5.0 to 6.0) | 11.0 (10.0 to 11.0)  5.0 (5.0 to 5.0)  6.0 (5.0 to 6.0) | 9.0 (8.0 to 11.0)  5.0 (4.0 to 5.0)  5.0 (4.0 to 6.0) | 10.0 (7.0 to 11.0)  4.0 (4.0 to 5.0)  6.0 (4.0 to 6.0) |
| **Loneliness: De Jong Gierveld (DJG) Loneliness Scale**  Categorised according to severity – N (%)  Not lonely (total score 0-2)  Moderately lonely (total score 3-8)  Severely lonely (total score 9-10)  Very severely lonely (total score 11) | 1 (3)  3 (10)  5 (17)  20 (69) | 0 (0)  2 (20)  3 (30)  5 (50) | 1 (3)  5 (13)  8 (21)  25 (64) | 1 (4)  8 (32)  7 (28)  9 (36) | 0 (0)  3 (30)  4 (40)  3 (30) |
| **Depression: Patient Health Questionnaire (PHQ-9)**  Total score - Mean (SD) | 21.6 (5.3) | 21.1 (4.5) | 21.4 (5.1) | 16.4 (6.8) | 18.8 (4.8) |
| **Anxiety: Generalized Anxiety Disorder Questionnaire (GAD-7)**  Total score - Median (IQR) | 19.0 (15.0 to 21.0) | 16.0 (11.0 to 18.0) | 17.5 (13.5 to 20.5) | 14.0 (10.5 to 17.5) | 13.5 (11.0 to 16.0) |
| **Wellbeing: Warwick-Edinburgh Mental Well-being Scale (WEMWBS)**  Total score | 26.5 (20.0 to 32.0) | 30.0 (24.0 to 34.0) | 28.0 (21.0 to 32.3) | 29.5 (23.0 to 34.5) | 31.0 (23.0 to 37.0) |
| **Social network: Lubben Social Network scale (LSNS6)**  Total score – Median (IQR)  Family subscale  Friends subscale | 7.0 (4.0 to 9.0)  4.0 (3.0 to 5.0 )  3.0 (1.0 to 6.0) | 11.5 (9.0 to 15.0)  5.5 (4.0 to 8.0)  6.0 (5.0 to 6.0) | 7.0 (6.0 to 12.0)  4.0 (3.0 to 6.0)  3.0 (1.5 to 6.0) | 7.5 (6.0 to 11.0)  5.0 (3.0 to 6.0)  3.0 (2.0 to 5.5) | 11.0 (6.0 to 15.0)  6.0 (3.0 to 8.0)  6.0 (4.0 to 8.0) |
| **Perceived social capital: Resource Generator UK (RGUK)**  Total score - Median (IQR)  Domestic resources subscale  Expert advice subscale  Personal skills subscale  Problem solving resources subscale  Human capital subscale | 9.5 (5.0 to 12.0)  3.0 (1.0 to 4.0)  2.5 (1.5 to 4.0)  1.0 (1.0 to 2.0)  2.0 (1.0 to 3.0)  2.0 (1.0 to 3.0) | 13.0 (8.8 to 18.3)  4.5 (2.0 to 5.0)  4.0 (3.0 to 7.0)  2.0 (2.0 to 3.0)  3.0 (2.0 to 4.0)  2.5 (1.0 to 3.0) | 10.0 (6.8 to 13.0)  3.0 (1.0 to 5.0)  3.0 (2.0 to 5.0)  2.0 (1.0 to 3.0)  2.5 (2.0 to 3.0)  2.0 (1.0 to 3.0) | 9.0 (6.0 to 12.3)  3.0 (2.0 to 4.0)  2.5 (1.5 to 4.5)  1.0 (1.0 to 2.0)  3.0 (2.0 to 3.0)  2.0 (1.0 to 4.0) | 13.0 (6.5 to 22.3)  5.0 (1.0 to 6.0)  5.0 (1.0 to 8.0)  1.5 (1.0 to 4.0)  3.5 (2.0 to 4.0)  3.0 (1.0 to 4.0) |
| **Time Budget Diary**  Total score – Mean(SD)  Activities performed alone subscale  Some contact with others subscale  Extensive contact with others subscale | 32.7 (9.6)  11.0 (8.5 to 14.5)  5.0 (2.0 to 9.5)  2.0 (0.5 to 4.5) | 38.4 (11.2)  11.0 (6.5 to 14.0)  3.5 (0.0 to 9.3)  2.0 (0.0 to 8.0) | 34.3 (10.2)  11.0 (8.0 to 14.0)  5.0 (2.0 to 9.0)  2.0 (0.0 to 5.0) | 36.9 (12.9)  8.0 (1.8 to 10.8)  8.5 (4.3 to 14.3)  1.0 (0.0 to 4.0) | 35.0 (14.4)  6.0 (3.0 to 6.0)  7.0 (6.0 to 13.0)  1.0 (0.0 to 2.5) |
| **Recovering Quality of Life Questionnaire (ReQoL-10)**  Total score - Median (IQR) | 9.0 (4.0 to 14.0) | 9.5 (5.0 to 15.0) | 9.0 (4.5 to 14.0) | 14.5 (8.0 to 19.0) | 13.5 (10.0 to 19.0) |
| **EuroQol Health Questionnaire (EQ-5D-5L) Index value** – Mean (SD) | 0.283 (0.40) | 0.400 (0.24) | 0.311 (0.37) | 0.472 (0.33) | 0.453 (0.236) |
| **Self-rated Health using EQ-Visual Analogue Scale (EQ VAS) -** Median (IQR) | 35.0 (29.0 to 50.0) | 47.5 (30.0 to 50.0) | 40.0 (30.0 to 50.0) | 40.0 (30.0 to 60.0) | 52.5 (35.0 to 60.0) |
| **Accepted for acute treatment in past 6 months (hospital or community crisis care**) – N (%) | 6 (20) | 0 (0) | 6 (15) | 5 (20) | 1 (10) |
| **Days in acute care -** Median (IQR) | 0.0 (0.0 to 0.0) | 0.0 (0.0 to 0.0) | 0.0 (0.0 to 0.0) | 0.0 (0.0 to 0.0) | 0.0 (0.0 to 0.0) |
| **Admitted to hospital** – N (%) | 1 (3) | 0 (0) | 1 (3) | 0 (0) | 1 (10) |
| **Inpatient bed days -** Median (IQR) | 0.0 (0.0 to 0.0) | 0.0 (0.0 to 0.0) | 0.0 (0.0 to 0.0) | 0.0 (0.0 to 0.0) | 0.0 (0.0 to 0.0) |
| **Community service kept appointments -** Median (IQR) | 6.5 (3.0 to 11.0) | 6.5 (1.0 to 17.0) | 6.5 (3.0 to 11.0) | 3.5 (2.0 to 10.0) | 8.0 (1.0 to 10.0) |
| **Community service missed appointments -** Median (IQR) | 0.0 (0.0 to 1.0) | 0.0 (0.0 to 1.0) | 0.0 (0.0 to 1.0) | 0.0 (0.0 to 1.0) | 1.5 (0.0 to 3.0) |
| **Missed one or more community service appointments** – N (%) | 11 (44) | 4 (44) | 15 (38) | 10 (33) | 6 (60) |

*IQR=Interquartile range

There were more missing data at baseline in the intervention than the control group for missed appointments (n=5 vs n=1; 17% vs 10%) and TBD (n=3 vs n=0; 10% vs 0%).  For all other outcomes at baseline and among participants who completed the follow up interview there was a maximum of 1 or 2 missing values per study arm, mostly in the intervention group, and missing data did not exceed 10% in either arm. The denominator for percentages is the number of non-missing values.

**Effect sizes for primary outcomes**

There are two candidate primary outcome variables for a future definitive trial, loneliness (measured using the 11-item De Jong Gierveld Loneliness Scale) and depression (measured with the PHQ-9). As planned in the protocol, we present univariate linear regression to estimate an (unstandardized) effect size detailing the mean difference between the groups on the PHQ-9 measure. We have not estimated an effect size for the DJG Loneliness Scale due to the highly skewed nature of the data.

Patient Health Questionnaire (PHQ-9) Total Score: Unadjusted Difference: -2.36, 95% CI: -7.17 to 2.45, p=0.326

Patient Health Questionnaire (PHQ-9) Total Score: Adjusted Difference: -2.54, 95% CI: -6.53 to 1.44, p=0.203

**Process Evaluation**

**Table 3: Take-up of the intervention**

|  | **Site 1 (N=15)** | **Site 2**  **(N=15)** | **Overall**  **(N=30)** |
| --- | --- | --- | --- |
| **Intervention Take-up Rate – N (%)**  **(Treated per protocol: min. 3 meetings)** | 14 (93.3) | 10 (66.7) | 24 (80.0) |

**Table 4: Attendance at meetings with Navigators**

|  | **All Intervention Participants (N=30)** | **Treated per Protocol**  **(N=24)** |
| --- | --- | --- |
| **Number of sessions attended (max 10) – Median (IQR)** | 6.5 (4.8 to 9.0) | 7.5 (5.3 to 9.8) |
| **Attended any meet-up group – N (%)** | 12 (40.0) | 12 (50.0) |
| **Meet-up groups – N (%)** |  |  |
| Group 1 | 10 (33.3) | 10 (41.7) |
| Group 2 | 6 (20.0) | 6 (25.0) |
| Group 3 | 9 (30.0) | 9 (37.5) |

**Table 5: Treatment as usual – participants’ care coordination status**

|  | **Intervention (N=30)** | **Control (N=10)** | **All**  **(N=40)** |
| --- | --- | --- | --- |
| **Care Coordinated – N (%)** | 18 (60.0) | 5 (50.0) | 23 (57.5) |
| **Without care coordinator -– N (%)** | 8 (26.6) | 2 (20.0) | 10 (25.0) |
| **Discharged from mental health services – N (%)** | 4 (13.3) | 3 (30.0) | 7 (17.5) |

Participants attended a total of 186 individual sessions with a Community Navigator. Session logs were provided by Community Navigators for all 186 sessions. These data are used to describe the content of activity in sessions below.

**Figure 2: The activities reported by Community Navigators in session logs**

| Domain of Navigator activity | % of total reported activities which were of this type |
| --- | --- |
| Network mapping | 8% |
| Setting goals | 6% |
| Breaking goals down into small steps | 4% |
| Finding resources/community assets | 17% |
| Planning activity | 21% |
| Going to community activities | 13% |
| Trouble-shooting | 8% |
| Contacting existing friends/family | 3% |
| Reflecting on progress | 12% |
| Planning for the future | 8% |

**Table 6. Navigator activity (from Community Navigators’ session logs)**

|  | **Intervention Participants who received some help of this type (N=30)** |
| --- | --- |
| **Navigation Activity – N (%)** |  |
| Did *Network Mapping* at any point | 26 (86.7) |
| Did *Setting Goals* at any point | 21 (70.0) |
| Did *Breaking goals down into small steps* at any point | 14 (46.7) |
| Did *Finding Resources/Community Assets* at any point | 24 (80.0) |
| Did *Preparing/planning activity* at any point | 28 (93.3) |
| Did *Going to Community Events* at any point | 20 (66.7) |
| Did *Trouble Shooting* at any point | 18 (60.0) |
| Did *Contacting existing friends/family* at any point | 5 (16.7) |
| Did *Reflecting on progress* at any point | 21 (70.0) |
| Did *Planning for the future* at any point | 20 (66.7) |
|  | |
|  | **Sessions (N=186)** |
|  | **N (%)** |
| **Location of Sessions**  Participants’ Home  NHS Premises  Community Location | 65 (34.9)  2 (1.1)  119 (64.0) |
| **Contact with other people**  Family Member  Other Study participants  Mental Health Professionals  Any Other People  No other contact apart from the navigator | 8 (4.3)  12 (6.5)  6 (3.2)  43 (23.1)  117 (62.9) |

**Use of the Navigation budget**

A budget of up to £100 per participant was available to support social activity agreed between the participant and their navigator. Nine out of 30 intervention group participants used the available budget to support their social activity. These nine used a total of £389. In total, only £389 out of the planned navigation budget of £3,000 was used.

**Table 7: Use of the study Navigation budget**

|  | **How much of it was spent?** |
| --- | --- |
| **Participant 1** | £64.00 |
| **Participant 2** | £89.00 |
| **Participant 3** | £34.00 |
| **Participant 4** | £10.00 |
| **Participant 5** | £32.00 |
| **Participant 6** | £20.00 |
| **Participant 7** | £100.00 |
| **Participant 8** | £20.00 |
| **Participant 9** | £20.00 |

**Inter-rater reliability**

Data were compared from two groups of raters (3 navigators in 1 group, 30 participants in another group) reporting provision of 10 different types of support. This introduces both clustering and repeated measures in an unbalanced design – not all raters are rating all of the 10 items.  As a result, we decided to summarise agreement using simple descriptive statistics. Analyses were undertaken by DS and RJ using Stata 14.

42 Participant feedback calls were completed. There was 72.38 % overall agreement between Community Navigator and participant for the 10 categories of support.

1. 69.05% agreement for Network Mapping
2. 61.90% agreement for Goal Setting
3. 76.19% agreement for Breaking goals down into small steps
4. 73.81% agreement for Finding Resources
5. 45.24% agreement for Planning an Activity
6. 78.57% agreement for Going to an Activity
7. 73.81% agreement for Trouble Shooting
8. 85.71% agreement for Contacting Existing Friends and Family
9. 76.19% agreement for Reviewing Progress
10. 83.33% agreement for Planning for the future

Notes: Missing cases – There were 540 observations and 120 missing cases. The denominator for percentages is the number of non-missing cases.

**5. Adverse Events**

**Serious adverse events:** Two serious adverse events (SAE) were recorded for one control participant (hospital admission) and one intervention participant (suicide) during the trial.

**Adverse events:** There were 23 adverse events, all in the intervention group:

4 CRT admissions

1 acute day unit admission

6 CRT referrals – not taken on for care

4 A&E attendance (self-harm or suicidal ideas)

3 mild self-harm or over use of medication – no medical attention

1 self-referral to private rehab clinic

3 physical ill health (epilepsy or fainting)

1 victim of domestic violence

None of these were identified by involved clinicians and site Principal Investigators as study-related. These judgements were confirmed by the Independent Clinical Reviewer from the Trial Steering Committee. Potential reasons why all the adverse events identified involved participants in the intervention group include:

- There were 30 participants in the intervention group, and only 10 in the control group
- Adverse events for the control group were only reviewed through a screen of patient records; adverse events in the intervention group were also reported by the Community Navigators

**6. Co-production on the Community Navigator Study**

| i) At the proposal stage  This study was a collaboration between researchers from UCL and The McPin Foundation. We used McPin’s expertise in peer-research and involvement to plan service user and carer involvement in the study in two main ways:  a) We employed a peer-researcher with lived experience of mental health problems, employed and managed through McPin, as one of the two study researchers – to lead on service user recruitment, data collection and analysis for the qualitative strand of the study and to help coordinate the study co-production group.  b) We planned and costed a co-production group, involving service users with lived experience expertise, practitioners and study researchers working together to develop the study intervention and contribute to all aspects of the study. We planned to include six service user experts by experience for this co-production group.  The funding proposal was reviewed by three members of the McPin Foundation peer review panel, who had lived experience of complex depression and anxiety.  We also consulted practitioners in developing the funding proposal through consultations with staff and managers at the clinical service where the study intervention would take place, and by presenting the study at the Camden and Islington Social Care Research Forum. |
| --- |
| ii) During the set-up  The co-production group met six times in the first four months of the study to develop the study intervention. This intensive level of intervention allowed time for service user and practitioner members of the group to orientate themselves to ways of addressing loneliness. (An overview of the research literature and talks from external loneliness experts were provided.) It also ensured decisions could be taken within the co-production group, without holding up the study. Service user members of the co-production group contributed to making key decisions about the content of the intervention, developing its theory of change, creating the intervention manual and training programme, and considering study outcome measures and recruitment materials.  We took steps to ensure all coproduction group members could contribute fully and their voices were heard. Preparation time was costed for service users reading before meetings. Service user members co-chaired some meetings, which were otherwise co-facilitated by the peer researcher. Small group work, and a pro-active approach to varying who sat where and worked with whom, built group cohesion and encouraged active contribution.  The McPin peer-researcher led on developing participant information sheets and consent forms, and qualitative interview topic guides. |
| iii) During recruitment and programme delivery  The study peer-researcher led on recruiting service user participants for qualitative elements of the study. By identifying herself as someone with lived experience, she sought to make participants feel at ease and build trust, to ensure honest, candid interviews. She also worked closely with the other study researcher to address any difficulties with quantitative participant recruitment.  Two service user members of the coproduction group were part of a four-person shortlisting panel for the Community Navigators, who would deliver the study intervention. Three other service user members devised a series of case study scenarios and assessed Navigators at interview. Three service user coproduction group members contributed to training the Navigators, facilitating practice role plays and giving feedback. The study peer researcher also contributed to interviewing the Navigators, and selection decisions were made jointly. A study researcher left after the recruitment period: one service user member of the coproduction group helped develop interview questions and conduct interviews for the new researcher.  The coproduction group continued to meet, and considered qualitative feedback from participants and Navigators from a preliminary testing phase of the study, before making decisions about adaptations to the intervention content and Navigator training. |
| iv) In data analysis and the dissemination of study findings  The study peer-researcher has led, and the coproduction group has been involved in, qualitative data analysis, ensuring decisions about key messages regarding participants’ experience of the study intervention are jointly developed and agreed. Service user members of the coproduction group have read interview transcripts, identified key themes within them, and thus contributed to developing an analysis coding frame.  Service user coproduction group members have attended an event organised by the study funder (NIHR School for Social Care Research) to discuss involvement in social care research, as ambassadors for our study.  Coproduction group members reviewed drafts of the published trial protocol paper, helped create blogs for the study website, and contributed to a prize-winning Enmesh conference poster on developing the intervention theory of change.  Co-production members also considered study quantitative and qualitative results and planned key findings and a dissemination strategy. The study peer researcher led writing up qualitative study findings. Co-production group members will contribute to writing papers, and may lead on writing up findings for service user publications. Service user members have helped us plan how to feed findings back to participants helpfully. |
